# Absence of detectable SARS-CoV-2 replication in ex vivo cultured cornea and cornea-derived epithelial cells

**DOI:** 10.1007/s00417-022-05776-6

**Published:** 2022-08-03

**Authors:** Tarek Bayyoud, Georgios Vavouras Syrigos, Natalia Ruetalo Buschinger, Johanna Wude, Ramona Businger, Dan Hu, Angelika Iftner, Sebastian Thaler, Michael Schindler

**Affiliations:** 1 Department of Ophthalmology, University Hospital Tübingen, Tübingen, Germany; 2 Institute for Medical Virology and Epidemiology of Viral Diseases, University Hospital Tübingen, Tübingen, Germany

**Keywords:** SARS-CoV-2, Ocular surface, Cornea organ transplantation, Human cytomegalovirus, HCMV, COVID-19

## Abstract

**Purpose:**

To study the possibility of SARS-CoV-2 to infect human corneal cells and tissues under standard corneal culture conditions using explants of COVID-19 donors and primary cornea-derived epithelial cells.

**Methods:**

Cornea isolated from deceased COVID-19 donors was cultured for 4 weeks, and SARS-CoV-2 replication was monitored by qRT-PCR. Furthermore, primary corneal epithelial cells from healthy donors were cultured ex vivo and infected with SARS-CoV-2 and human cytomegalovirus (HCMV) as a control. Infection status was assessed by western blotting and reporter gene expression using green fluorescent protein–expressing viral strains. ACE2 and TMPRSS2 receptor expression levels in cornea and epithelial cells were assessed by qRT-PCR.

**Results:**

We did not detect SARS-CoV-2 replication in 10 corneas isolated from deceased COVID-19 patients and cultured for 4 weeks, indicating absence of infection under natural conditions. Furthermore, high-titer SARS-CoV-2 infection of ex vivo cultured cornea-derived epithelial cells did not result in productive virus replication. In contrast, the same cells were highly permissive for HCMV. This phenotype could potentially be explained by low ACE2 and TMPRSS2 transcriptional activity in cornea and cornea-derived epithelial cells.

**Conclusions:**

Our data suggest that cornea and limbal epithelial cells are refractory to productive SARS-CoV-2 infection. This could be due to the absence of robust receptor expression levels necessary for viral entry. This study adds further evidence to support the very low possibility of transmission of SARS-CoV-2 from an infected corneal transplant donor to a recipient in corneal organ cultures.

**Supplementary Information:**

The online version contains supplementary material available at 10.1007/s00417-022-05776-6.

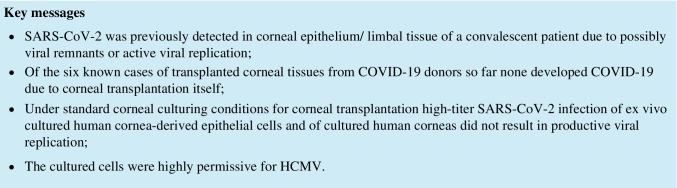



## Introduction

The COVID-19 (Coronavirus Disease 2019) pandemic poses challenges in ophthalmology and particularly eye banking during tissue harvesting and processing. The risk to infect transplant recipients with SARS-CoV-2 appears to be very unlikely considering the pathophysiology of the other two coronavirus epidemics of Severe Acute Respiratory Syndrome-Coronavirus-1 (SARS-CoV-1) and Middle East Respiratory Syndrome-Coronavirus (MERS-CoV). Moreover, former and recent Center for Disease Control (CDC) and Food and Drug Administration (FDA) guidelines do not assume a transmission of a respiratory virus through tissue transplantation including human cells, tissues, cellular, and tissue-based products supported by recent studies [1–3]. Nevertheless, the potential presence of viral entry factors in the cornea suggests that infection of human corneal tissue is at least possible [4, 5]. In addition, the documented isolation of SARS-CoV-2 from human tears and conjunctival swabs demands to exercise even further caution [6–8]. The current practice of largely excluding ocular tissue donations from COVID-19 positive cases or suspected COVID-19 cases leads to a significant temporary decline in the number of donors thereby exacerbating tissue shortage [9]. With regard to coronaviruses, especially SARS-CoV-2 as well as SARS-CoV-1 or MERS-CoV, no data have yet been published for detection or non-detection in corneal organ culture and cultured human corneas under standard conditions. At present, there are only a limited number of studies dealing with the detection of viruses in corneal organ culture in general and the transmissibility of viruses via corneal tissue [10–12]. Moreover, organ cultures under physiological conditions might potentially support the replication of SARS-COV-2 from corneal tissues of infected donors. This is relevant, as subclinical cases of COVID-19 might inadvertently be considered for tissue donation and transplantation in case of a non-SARS-CoV-2-associated cause of death. Thus, the possibility of transmission of SARS-CoV-2 via tissue culture media is highest for the group of asymptomatic SARS-CoV-2 donors. Furthermore, validated postmortem test kits are not yet available to be able to carry out a safe exclusion of SARS-CoV-2 infection for such tissue. Fortunately, of the 8 cases in which corneal tissue from infected donors was transplanted known to date in the United States only one developed COVID-19 attributed to community acquisition rather than via corneal transplantation [2]. In the present study, we assessed if SARS-CoV-2 replicates in cultured human full-thickness corneas of COVID-19 donors. We further investigated if cultured corneal epithelial cells are permissive for SARS-CoV-2 and used Human cytomegalovirus (HCMV) as positive control, as it is known to have a broad cell tropism for epithelial and endothelial cells.

## Methods

### Ethics

We adhere to all formal regulations according to the Declaration of Helsinki. We obtained informed consent and approval by the independent Ethics Committee (institutional review board) of the University Hospital Tübingen prior to commencing with the study (IRB# 642/ 2020BO2).

### Corneal tissue culture and behavioral procedures

We employed standardized culturing technique to prevent any kind of contamination and/or tissue damage. In addition, standardized quality controls were in place. In detail, the involved employees were trained and qualified to perform the tissue and cell culture techniques. The general guidelines governing the procedural facilities (including Biosafety Level 3, BSL-3), the hygiene and disinfection guidelines for corneal banking, and the guidelines for producing and labeling the stock solutions and/ or media were employed. This included identification number, volume/quantity, expiration date, item number, batch number and storage conditions. The preparation of the corneas was carried out with single-use, sterile instruments.

### Tissue extraction, preparation and cultivation

Ten corneas were extracted from COVID-19 postmortem donors (*N* = 5) after informed consent of next of kin according to the regulations of organ donation [13]. According to standard operating procedural guidelines for corneal transplantation, removal of conjunctiva was carried out. Trephination of corneo-scleral tissue was performed using globe holders (under aspiration with a 5-mL syringe). The separation of cornea from ciliary body was performed using Sautter tweezers. Finally, the tissue was placed in culture medium I (KM-1; Biochrome, #9016, Berlin, Germany). The average time of death to retrieval and to preservation was 21 and 31 h, respectively. Cornea-scleral discs were cultured for up to 4 weeks in T25 cell culture flasks. Every 2–3 days aliquots of the cell culture media were taken and stored at −80 °C for subsequent analysis. At the end of the culture period cornea were lysed with 600 μL RLT buffer for qRT-PCR analysis.

### Isolation and culture of human corneal epithelial cells

Human corneo-scleral rims were made available from the Eye Bank of the Department of Ophthalmology after obtaining informed consent. Epithelial cell cultures were obtained from 1.2 mm in diameter discs that were taken from the limbus under a microscope. Samples were incubated at 37 °C in 3 mg/mL dispase II dissolved in CnT-PR (CELLnTEC Advanced Cell Systems AG, Bern, Switzerland) for 1 hour. Subsequent incubation with 0.25% Trypsin/EDTA (Gibco, Invitrogen) for 10 min yielded single-cell suspensions, which were seeded in 24-well plates (Costar, Corning, NYSE, GLW). Human corneal epithelial cells (HCEC) were maintained in CnT-PR, 100 U/mL penicillin G, and 100 mg/mL streptomycin sulfate in 5% CO_2_-humidified environment. Cell characterizations were performed using epithelial specific corneal tissue marker (cf. Supplementary Information; SI; markers included AE5 (SI-1) and CK12 (SI-2) ). Medium exchange was done every 2–3 days. Types of plates used were 96-well plates (Corning, NY, #3596) with 10^4^ cells per well. First cell passages were employed.

### Cell lines

SARS-CoV-2 permissive colon cancer-derived epithelial cells Caco-2 and human lung cell line Calu-3 were cultured and used as described before [14, 15]. Furthermore, primary human foreskin fibroblast (HFF) to culture HCMV were employed [16]. As positive control for ACE2 and TMPRSS2 receptor expression, we employed the A549 lung cell lines engineered to express both entry receptors and cultured them as described [15].

### Viruses

All experiments associated with SARS-CoV-2 were conducted in BSL-3 laboratory. The recombinant infectious SARS-CoV-2 clone expressing mNeonGreen (icSARS-CoV-2-mNG) [17] was obtained from the World Reference Center for Emerging Viruses and Arboviruses (WRCEVA) at the UTMB (University of Texas Medical Branch). SARS-CoV-2 WT (SARS-CoV-2 200325_Tü1) was isolated from a patient sample, and the variant identity was confirmed by next-generation sequencing of the entire viral genome [14]. Human cytomegalovirus strain used is the TB40E lab strain. For the infection experiments, the WT and the delUL16-eGFP HCMV were thawed from frozen viral stocks and directly added to cells [16].

### Infection experiments

Corneal epithelial cells that were differentiated in 24-well plates were infected with SARS-CoV-2 clinical isolate 200325_Tü1 at MOI (multiplicity of infection) 10, with the icSARS-CoV-2-mNG at MOI 10 or mock-infected. For SARS-CoV-2, stocks were titered on Caco-2 epithelial cells and infectivity further confirmed on Calu-3 lung cell lines, as previously described [14, 15]. For the HCMV infection, cells were infected with HCMV viral stocks at MOI 10 or mock infected. HCMV virus stocks were titered on human foreskin fibroblasts (HFF) [16]. Cells were monitored, and images were taken 24 and 48 h post-infection using the image reader Cytation3 (Biotek).

### Western blot

Forty-eight hours post infection (hpi) with SARS-CoV-2 or HCMV, cells were harvested and lysed using RIPA buffer (10 mM Tris-HCl pH 7.4, 150 mM NaCl, 1% TritonX-100, 0.1% Na-deoxycholate, 0.1% SDS, 1 mM EDTA, 0.5 mM EGTA, Protease Inhibitor Cocktail Tablets, EDTA-Free (Sigma)). Samples were analyzed by SDS-PAGE and transferred to a nitrocellulose-membrane by wet transfer and blocking in 5% milk in TBS for 1 h at room temperature. Detection of SARS-CoV-2 proteins was performed using the serum from a hospitalized convalescent donor in a 1:1000 dilution and a goat anti-Human IgG secondary antibody (LI-COR) in a 1:15,000 dilution. Detection of HCMV-proteins was performed using the mouse anti-cmv pp52 (10D8, Virusys corporation, 1:1000) and the mouse anti-cmv p28 (CH19, Virusys Corporation, 1:100) antibodies. Both primary antibodies were detected using the IRDye® 680RD Goat anti-Mouse IgG Secondary Antibody (LI-COR, 1:15,000 dilution). As house-keeping protein actin and tubulin were used, which was detected using monoclonal mouse anti-actin and tubulin antibodies: anti-actin (#A3853, Sigma) 1:1000 dilution in 5% milk in TBST 1 h at RT or overnight in 4 °C; anti-tubulin (# PA5-22060, Invitrogen) 1:1000 dilution in 5% milk in TBST 1 h at RT or overnight at 4 °C.

### SARS-CoV-2, ACE2, and TMPRSS2 qRT-PCR

Corneal epithelial cells were harvested and lysed using RLT buffer w/o ß-Mercaptoethanol (Qiagen), and RNA was extracted using the RNeasy Mini Kit (Qiagen). Afterwards, cDNAs of the cells were generated using the QuantiTect Reverse Transcription Kit (Qiagen), and samples were prepared for qRT-PCR using the Luna Universal qPCR Master Mix (New England Biolabs), following the dsDNA-binding dye procedure. Specific primers for ACE2 (forward: GATGCCTCCCTGCTCATTTG, reverse: AACTTCTCGGCCTCCTTGAA) and TMPRSS2 (forward: AGGACGAGAATCGGTGTGTT, reverse: GGATCCGCTGTCATCCACTA) were used, and the qPCR was conducted in Lightcycler 480 II (Roche), using the SYBR green protocol as suggested by the manufacturer and as described previously, including the specific primers for GAPDH [15]. After 4 weeks of culturing, corneas were stored in 600 μL RLT buffer w/o ß-Mercaptoethanol (Qiagen), and supernatants were mixed at a ratio of 1:1 with 300 μL RLT buffer. RNA extraction and SARS-CoV-2 qRT-PCR were done as described [13].

### Basic demographic and clinical information of COVID-19 donors

The age of the COVID-19 donors ranged from 74 to 89 years (mean: 80 years; 1 woman and 4 men, each patient/next of kin consented to donate both eyes, no globe had to be excluded due to medical reasons). Medical history included arterial hypertension in all and diabetes mellitus in one patient. All donors were on anti-hypertensive treatment at time of admission. In addition, one patient was on an anti-hyperglycemic regimen. The detailed drug history included three patients on angiotensin-converting enzyme (ACE) inhibitor class of medication, 1 patient on an angiotensin-receptor blocker (ARB), and another on the anti-hyperglycemic biguanide agent metformin. The mean time of hospitalization prior to death was 15 days (±12.9 SD; range: 1–32 days). All patients presented with unspecific symptoms and progressed to the full picture of COVID-19 pneumonia. Pharyngeal swabs and bronchoalveolar lavage fluid were tested positive for SARS-CoV-2-RNA by qRT-PCR. Co-infections by herpes simplex virus, cytomegalovirus, respiratory syncytial virus, parainfluenza, and influenza were excluded through qRT-PCR. The type of care included supportive, respiratory intubation, and machine-assisted support (MAS). MAS involved extracorporeal membrane oxygenation (ECMO) and continuous veno-venous hemofiltration (cVVH). Supportive care was administered to all patients, respiratory ventilation was needed in 4 patients, and MAS finally in 3 patients (1·ECMO and 2·cVVH). Organ system involvement was extensive in all cases. This encompassed the respiratory, uro-genital, and gastro-intestinal systems. The respiratory system was involved in all cases leading to acute respiratory distress syndrome (ARDS) in all patients. ARDS was complicated by myocardial infarction in 3, pleural effusion in 2, and atrial fibrillation in 1 patient. A life-threatening organ dysfunction was diagnosed in all patients extending to involve the genito-urinary and gastro-intestinal systems. Acute kidney failure was observed in all and acute liver failure in 2 patients. Multiorgan dysfunction syndrome (MODS) was diagnosed in 3 patients. Laboratory analysis revealed a reduced hemoglobin concentration (*N*=4) and a combined picture of leukocytosis-lymphopenia (*N*=2). All COVID-19 patients were in active infection at time of demise [13].

## Results

### Absence of detectable SARS-CoV-2 replication in long-term cultured cornea from COVID-19 donors

**Fig. 1:**
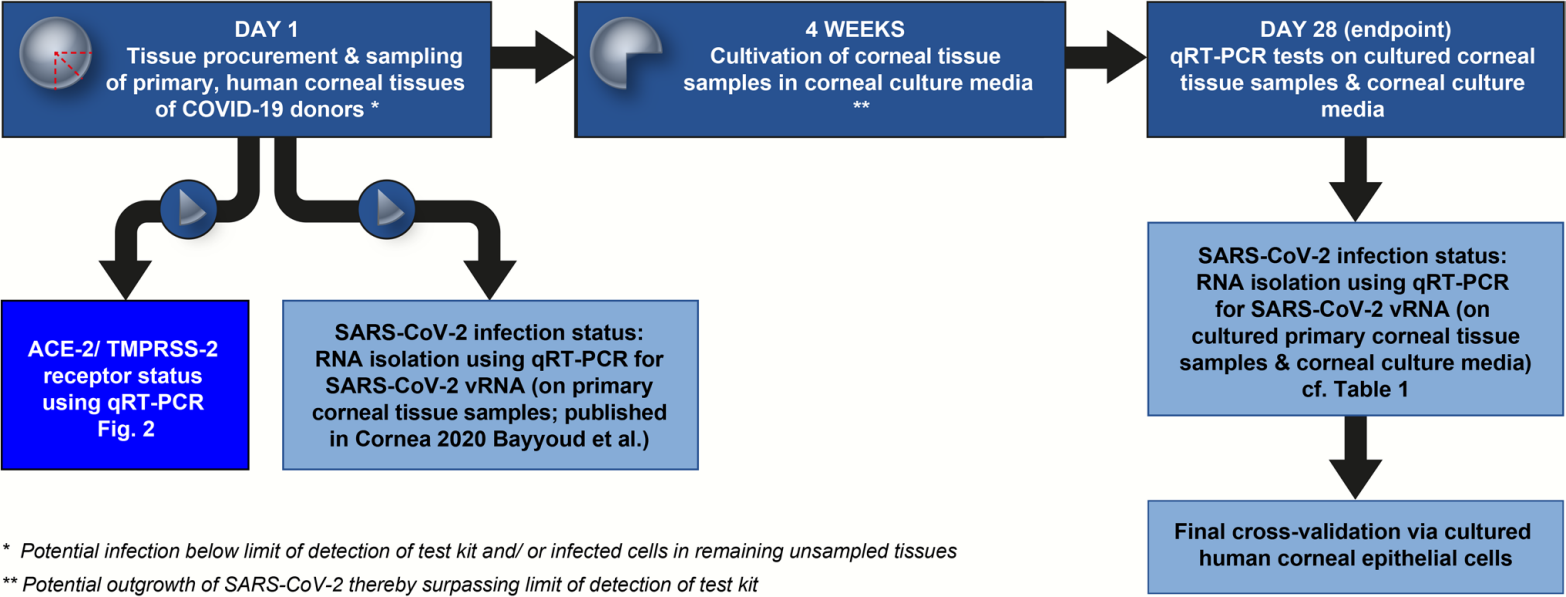
Experimental layout and study plan. Primary human corneal tissues were prepared from deceased COVID-19 patients and assessed for SARS-CoV-2 infection status by qRT-PCR (Bayyoud et al. 2020). The same RNA extraction was used to quantify ACE2/TMPRSS2 mRNA levels in these tissues. Furthermore, full thickness corneas with associated tissues were cultured for 4 weeks according to cornea organ culture conditions. RNA was extracted from cell culture media and tissue samples to analyze them for vRNA and hence SARS-CoV-2 replication by qRT-PCR. Furthermore, cornea donations from healthy donors were used to generate primary cornea-derived epithelial cells and subsequently infected with SARS-CoV2 and HCMV as a control

We previously excluded the presence of SARS-CoV-2 in various eye tissues from patients who died from COVID-19 immediately after enucleation [13, 18]. While we did not detect SARS-CoV-2 by qRT-PCR in these tissues, there is a constant threat of a low number of infected cells possibly remaining under the limit of detection. Hence, a sustained viral replication under standard culture conditions might be a risk. Therefore, we designed a study plan allowing us to detect viral infection in ex vivo cultured primary corneal tissue. In parallel, we assessed SARS-CoV-2 entry receptor expression (Fig. 1).

**Fig. 2:**
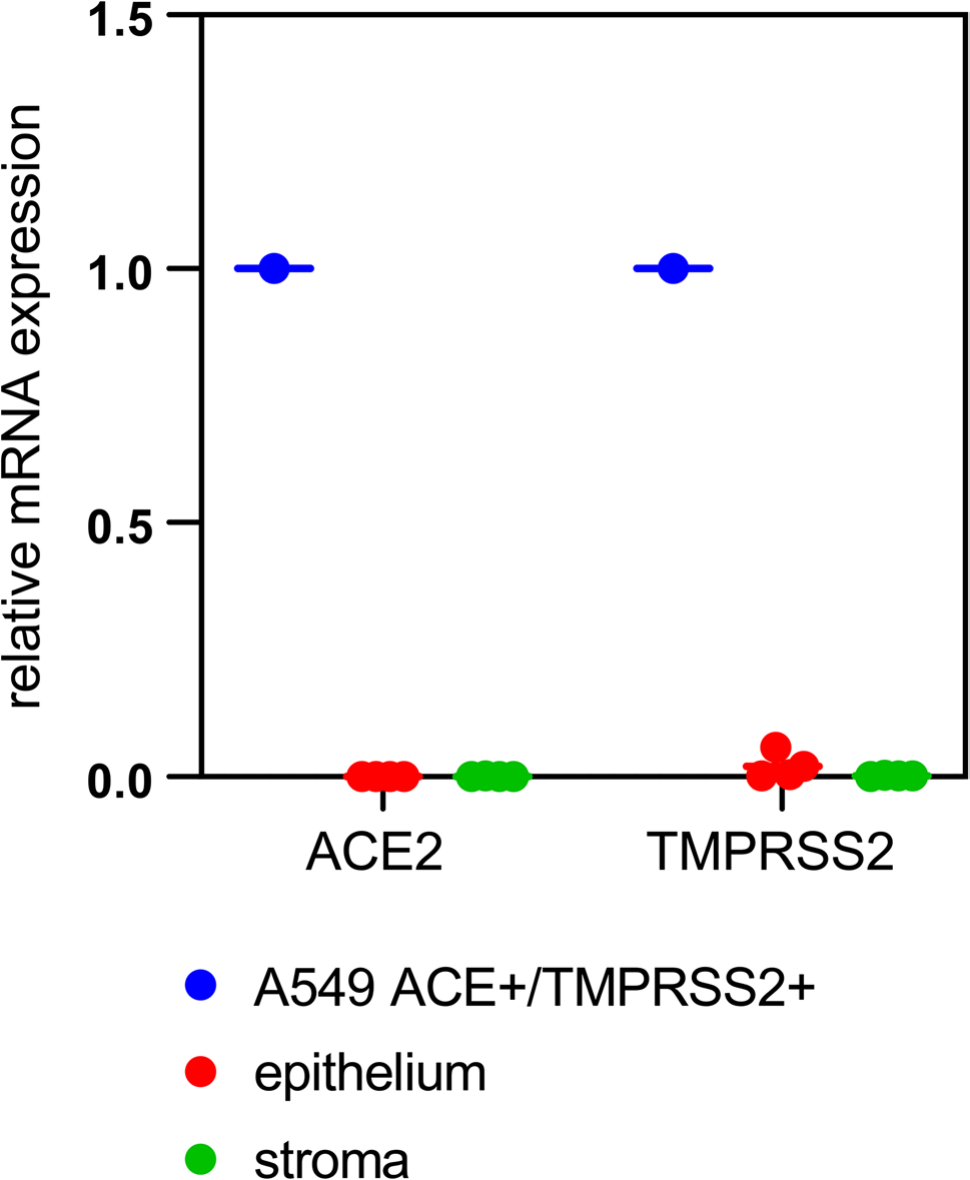
Undetectable ACE2 and TMPRSS2 mRNA levels in primary cornea tissue. Cornea tissue (stroma and epithelium) from deceased COVID-19 donors was prepared as described (see M&M (Material and Methods) section and [13]) and used to quantify ACE2 and TMPRSS2 mRNA levels by qRT-PCR. Data is normalized to transcriptional expression of the housekeeping gene (GAPDH), and ACE2 and TMPRSS2 mRNA levels in the transgenic cell line A549-ACE2/TMPRSS2 were set to 1. Tissue samples from 5 donors were analyzed

The full-thickness corneas and the associated corneal tissue, which we used to directly assess SARS-CoV-2 infection status after enucleation, were cultured for an additional 4 weeks. The standard protocol for culturing cornea for transplantation purposes according to current regulations was used [19, 20]. Aliquots of the cell culture supernatants were taken in 2- to 3-day intervals over a period of 4 weeks (Fig. 1). At the end of the culture period, RNA was extracted from the culture media, the full-thickness corneas, and the associated tissue. We then performed qRT-PCR to detect SARS-CoV-2 RNA and thus signs of viral replication over the 4-week culture period. None of the samples gave a signal above the limit of detection (Table 1), indicating the absence of SARS-CoV-2 infection and replication in cornea and associated tissues of deceased COVID-19 patients. Expression of ACE2 and TMPRSS2, the most important SARS-CoV-2 entry receptors, was reported on cornea and ocular tissue based on immunohistochemistry [4]. We did not find any evidence for SARS-CoV-2 replication in our tissue culture. Additional qRT-PCR to measure ACE2 and TMPRSS2 mRNA levels in corneal tissue obtained immediately after enucleation was performed. Established A549-cells, which stably express ACE2 and TMPRSS2, were used as a positive control [15]. No significant amounts of ACE2 or TMPRSS2 mRNA were detectable in any of the primary corneal tissue samples derived from either stroma (which also contains the endothelium) or epithelium (Fig. 2). Our data indicate that primary cultured corneal organ transplants fail to undergo infection and low-level replication under standard corneal bank culture conditions. This might be explained by the absence of a robust SARS-CoV-2 receptor expression based on transcriptional activity.

**Table 1:** COVID-19 Postmortem Cultured Donor Tissues, Corneal Culture Media and SARS-CoV-2 qRT-PCR Results

Ocular Tissue/Fluid ID	Type of Ocular Tissue/Fluid	RNA Yields (in ng/μl)	qRT-PCR for SARS-CoV-2 RNA
1C01	Cultured cornea	28.62	vRNA undetectable
1M01	Corneal culture media	29.45	vRNA undetectable
1C02	Cultured cornea	28.69	vRNA undetectable
1M02	Corneal culture media	32.35	vRNA undetectable
2C01	Cultured cornea	28.81	vRNA undetectable
2M01	Corneal culture media	31.72	vRNA undetectable
2C02	Cultured cornea	29.45	vRNA undetectable
2M02	Corneal culture media	31.79	vRNA undetectable
3C01	Cultured cornea	29.90	vRNA undetectable
3M01	Corneal culture media	32.12	vRNA undetectable
3C02	Cultured cornea	29.39	vRNA undetectable
3M02	Corneal culture media	31.95	vRNA undetectable
4C01	Cultured cornea	28.27	vRNA undetectable
4M01	Corneal culture media	32.43	vRNA undetectable
4C02	Cultured cornea	28.94	vRNA undetectable
4M02	Corneal culture media	31.68	vRNA undetectable
5C01	Cultured cornea	29.82	vRNA undetectable
5M01	Corneal culture media	30.00	vRNA undetectable
5C02	Cultured cornea	28.47	vRNA undetectable
5M02	Corneal culture media	30.16	vRNA undetectable

qRT-PCR, quantitative reverse transcriptase-polymerase chain reaction (S-/E-genes, positive/internal controls); vRNA, viral RNA; cultivation period of 4 weeks.

### Ex vivo cultured primary cornea-derived epithelial cells are not permissive for SARS-CoV-2 infection

In addition to monitoring SARS-CoV-2 infection in corneal tissue from deceased COVID-19 patients, we set out to establish a system for high-titer ex vivo infection of primary cornea-derived epithelial cells from healthy donors. For this purpose, corneal explants were cultivated in 24-well plates over a period of 2 weeks [21, 22]. After outgrowth of cornea-derived epithelial cells in the culture well, we performed high-titer infection with SARS-CoV-2 and human cytomegalovirus (HCMV), a virus with a broad tropism including endothelial and epithelial cells. Importantly, the titer of all virus preparations was tightly controlled in parallel to ensure virus stocks are infectious and permissive for Caco-2 epithelial and Calu-3 respiratory lung cell lines (SARS-CoV-2) and HFF (human foreskin fibroblasts, HCMV). We employed a clinical primary SARS-CoV-2 isolate (“WT”-strain) [14] as well as a modified version expressing the fluorescence reporter mNeonGreen instead of ORF7A [17]. Similarly, we used an endotheliotropic HCMV-strain (TB40E) with and without GFP reporter [16].

**Fig. 3:**
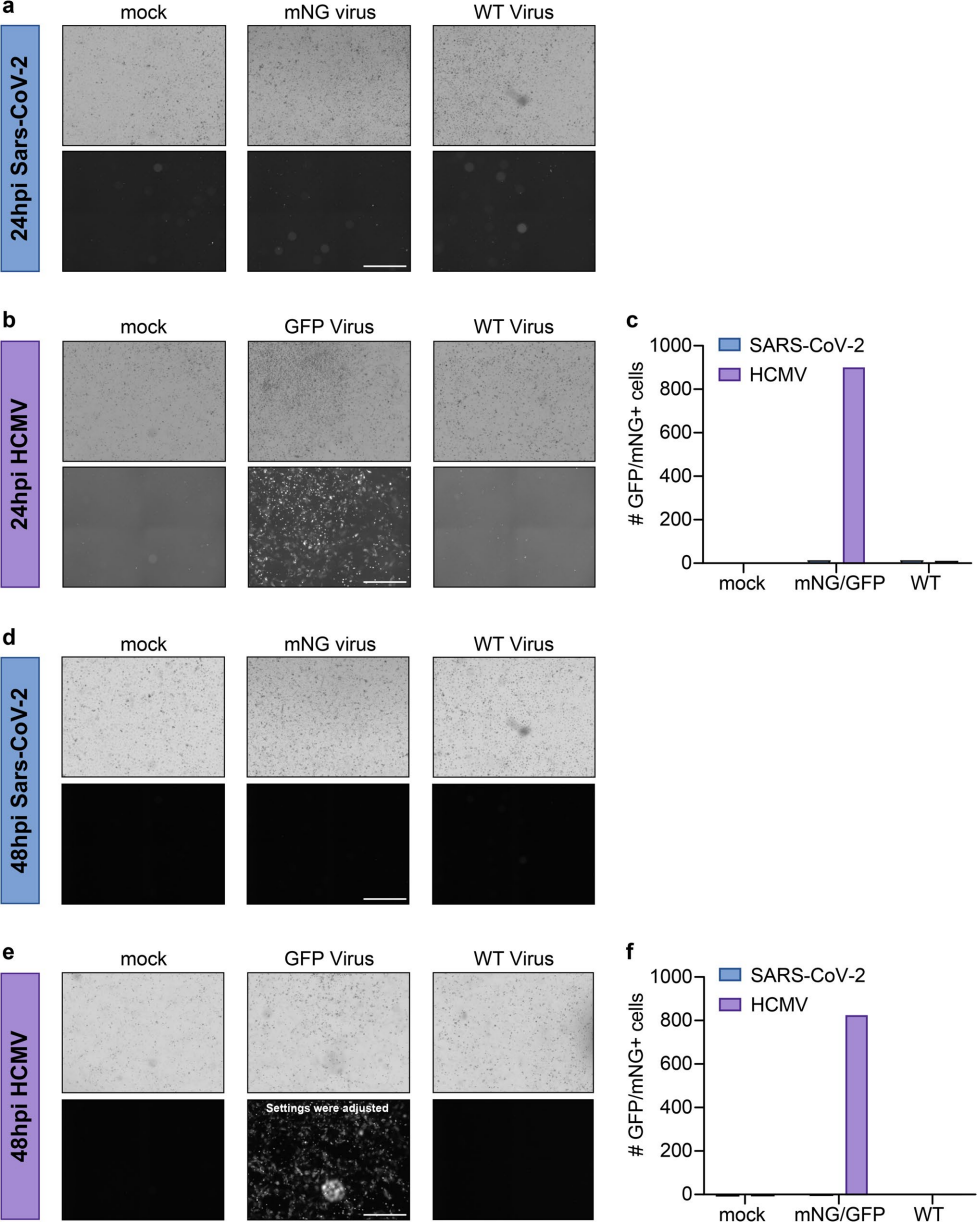
Primary cornea-derived epithelial cells are not permissive for SARS-CoV-2 infection. Corneal epithelial cells were differentiated in 24-well plates and (**a**, **d**) infected with a high-titer virus stock of a mNeonGreen-expressing SARS-CoV-2 isolate or a patient-derived primary strain. Alternatively (**b**, **e**) cells were infected with HCMV-GFP or the corresponding WT-strain. Images were taken 24 hpi (**a**, **d**) and 48 hpi (**b**, **e**) and the total amount of chromophore expressing cells per image counted by automated microscopy (**c**, 24 hpi and **f**, 48 hpi). Imaging was done with an objective at 4-fold magnification. The scale bar indicates a distance of 1 mm. We confirmed this data with corneal cells from one additional donor

We then monitored virus replication and propagation by fluorescence microscopy (Fig. 3). At 24 hpi, we could not detect any fluorescence when corneal epithelial cells were infected with SARS-CoV-2-mNG, or signs of a cytopathic effect when infected with WT SARS-CoV-2 (Fig. 3a). In contrast, a high proportion of cells were already GFP-positive after infection with HCMV-GFP (Fig. 3b and quantification Fig. 3c). Assuming that SARS-CoV-2 might have delayed viral replication kinetics compared to HCMV, we monitored the infection for an additional 24 h (48 hpi). However, even at this point in time, fluorescent cells were only observed in the case of an infection with HCMV, but not with SARS-CoV-2 (Fig. 3d–f).

**Fig. 4:**
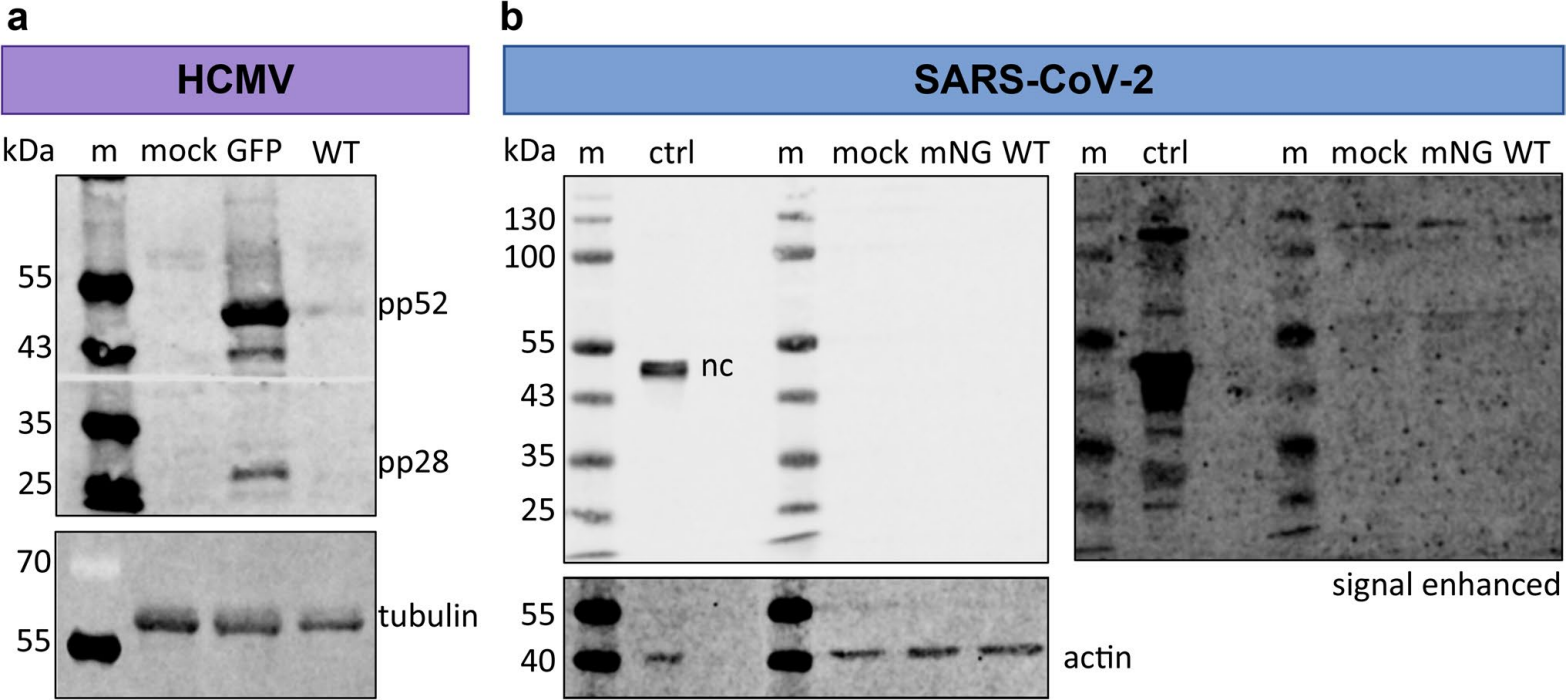
Primary cornea-derived epithelial cells support HCMV but not SARS-CoV-2 replication. Corneal epithelial cells were infected as described in M&M, and cell lysates for western blot analysis were prepared 48 hpi. Western blotting was performed to detect (**a**) HCMV or (**b**) SARS-CoV-2 viral protein expression. As a positive control for SARS-CoV-2 infection (“ctrl”), we used a lysate of SARS-CoV-2 infected Caco-2 cells at 48 hpi. We confirmed this data with corneal cells from one additional donor. m: marker; nc: SARS-CoV-2 nucleocapsid

To independently assess viral infection and cell replication, we prepared lysates of corneal epithelial cells for western blot analysis of viral protein expression. Human cytomegalovirus proteins were detected in the cell lysates of TB40E-GFP infected cells and to a lesser extent in the WT-infected cells (Fig. 4a). In contrast, as expected from fluorescence microscopic analysis, no SARS-CoV-2 protein was detectable in either SARS-CoV-2-mNG or WT-infected corneal epithelial cells (Fig. 4b). As an infection control, we used lysates from SARS-CoV-2 WT infected Caco-2 cells, a colon-derived epithelial cell line, in which the SARS-CoV-2 nucleoprotein was readily detectable. In summary, while primary cornea-derived epithelial cells appear to be permissive for HCMV infection, SARS-CoV-2 does not efficiently replicate in these cells.

**Fig. 5:**
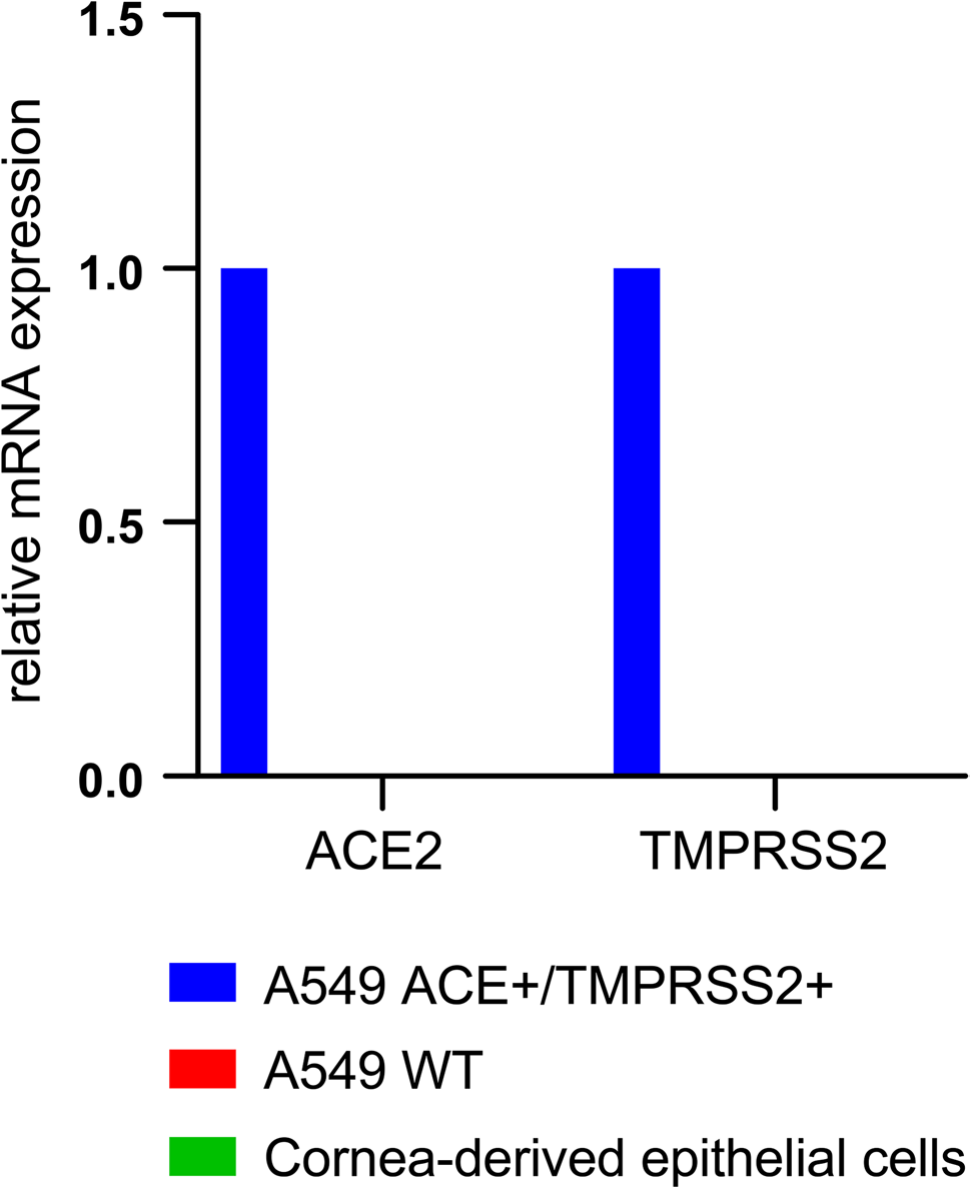
Undetectable ACE2 and TMPRSS2 mRNA-levels in primary cornea-derived epithelial cells. Corneal epithelial cells were infected as described in M&M, and RNA was extracted 48 hpi. We prepared cDNA to quantify ACE2 and TMPRSS2 mRNA levels by qRT-PCR. Data is normalized to transcriptional expression of the housekeeping gene (GAPDH), and ACE2 and TMPRSS2 mRNA levels in the transgenic cell line A549-ACE2/TMPRSS2 were set to 1. As additional negative control, we employed the non-transgenic parental A549 lung cell line. We confirmed this data with corneal cells from one additional donor

In addition, we determined steady-state mRNA levels of the SARS-CoV-2 receptors ACE2 and TMPRSS2 in the primary corneal epithelial cells. Consistent with our previous results using cornea-derived stroma and epithelium from postmortem eye tissue, we found no evidence for robust receptor expression based on transcriptional activity (Fig. 5). We therefore conclude that one of the possible reasons why SARS-CoV-2 does not replicate in corneal tissue is due to the lack of robust viral entry receptor expression.

## Discussion

In our study, we demonstrated that SARS-CoV-2 has an undetectable level of replication in long-term cultured corneas of COVID-19 donors and does not infect and replicate primary cornea-derived epithelial cells. Consistent with this is the lack of evidence for robust receptor expression of viral entry factors in corneal tissues and epithelial cells based on transcriptional activity. These findings are in line with the absent infection status of SARS-CoV-2 on unfixed, fresh corneal tissue [13] and the undetectable transcription activity of viral entry factors on them.

The potential risk of virus transmission from corneal transplantation has been addressed and discussed in several studies [1, 2, 4, 5, 9, 10, 13, 18], and there is evidence available to support the potential risk of transmission, as well as evidence against it. No transmission of SARS-CoV-2 from the donor to the recipient has been documented in transplantation procedures carried out to date [2]. However, the presence of SARS-CoV-2 in the tear film [7, 8] and possible steady-state receptor expression on the ocular surface and in the cornea [23, 24] indicate the ability of SARS-CoV-2 to infect ocular tissues in vitro and in vivo. Note that in an experimental setting, the relative expression of SARS-CoV-2 replication was lowest in the cornea (*n*=4) compared to other ocular tissues [25]. Also, the presence of SARS-CoV-2 in human postmortem ocular tissue supports the risk of donor-to-recipient transmission, although there is no evidence of active infection or virus replication on ocular tissues [4, 23, 26–29]. A very recent study on the expression of ACE2 and TMPRSS2 receptors in human limbal epithelial cells deserves special attention in the management of patients affected by COVID-19-associated eye disease [30]. On the contrary, the proven virucidal effect of povidone-iodine on SARS-CoV-1 and SARS-CoV-2, the inability of SARS-CoV-2 to infect and replicate in human corneal explants, the absence of the virus in the human postmortem eye tissue, and the documented lack of transmission of SARS-CoV-2 by corneal transplantation from infected donors reinforce the very low possibility or even impossibility of transmission by classical organ culture techniques and tissue [1, 13, 31–33]. We present results that may explain the inability of SARS-CoV-2 to infect corneal cells and cornea under standard corneal bank culture conditions. The transcription profile indicates a lack of robust receptor expression that may be insufficient for sustained viral replication.

We assessed SARS-CoV-2 replication and mRNA-based receptor levels in 4-week cultured full-thickness corneas from deceased COVID-19 patients. In addition, we used primary cornea-derived epithelial cells cultured ex vivo. This model system was also not permissive for SARS-CoV-2 infection and replication when challenged with a high titer virus, and again we could not detect mRNA expression of ACE2 and TMPRSS2. We have carried out extensive checks. In detail, transgenic A549-lung cells engineered to express ACE2 and TMPRSS2, control lysates of Caco-2 cells 48 hpi with SARS-CoV-2, and most importantly we checked the permissivity of our ex vivo cell culture system for infection per se validated by infection with HCMV. HCMV is a virus with a relatively broad cell tropism that includes fibroblast, endothelial, and epithelial cells [34]. By co-infection with HCMV, whether expressing GFP or not, we could show that cornea-derived epithelial cells are highly permissive for HCMV infection. While this finding is primarily an important control, there is a further concern that the eye in general, and the cornea in particular, could serve as a reservoir for HCMV that could be transmitted during transplantation. Although it is clear that HCMV can spread to the retina and cause pathologies [35], our data suggest that the cornea may be a new and not yet established entry site for HCMV. While this is currently speculative, this concern could be addressed and debated in the future.

The main limitations of the current study could be the clinical courses of the COVID-19 organ donors (e.g., a protracted clinical course to eventual death could impact the probability of detecting virus growth in cell cultures), the lack of expression of the entry receptor and infectivity, which may be altered or compromised by post-mortem sampling, systemic disease, concomitant systemic infections, immune status, and donor age. In addition, a variety of parameters, including the type of preservation, age of the sample, and cultivation conditions, which may have an impact on the results, need to be considered. The protracted course of the COVID-19 patients with mostly intensive care stays until the maximum therapy options have been exhausted, i.e., late phase of the disease with a possible convalescent stage of the ocular mucosa, could partially contribute to the results. Entry receptor expression can be affected by the elapsed times from death to collection and from death to preservation, in addition to the media and solutions that have come into contact with the cells and tissues during collection, transport, preparation, and culture. This could reconcile our results with previous work demonstrating entry receptors in ocular surface tissues. In addition, immature eye cells or cell models were used in some studies [36]. The results may therefore differ with mature ocular cell lines or cells from a different cell niche. A very interesting case report by Kuo et al. describes keratouveitis in a convalescent patient with detectable SARS-CoV-2 RNA in the corneal epithelium [37]. Whether the result was due to active viral replication or was caused by viral remnants was not yet clear [37]. A recent publication reports successful infection of the corneal epithelium (IOVS abstract of 2021 Vol. 62, Issue 8 by Singh et al.). Other potential factors influencing ocular surface infection and thus transmission via corneal transplant cultures include effective mechanical clearance of the virus, vigorous immune cell-mediated clearance of the virus, immune-mediated responses to attenuate inflammation, and adequate levels of exposure to an adequate viral load [23]. These factors could also explain the rarity of overt viral conjunctivitis in patients with COVID-19 [23]. Finally, the outcome of the absence or lack of detection of SARS-CoV-2 in the culture media flasks of SARS-CoV-2 donor tissues at the end of the legally allowed culturing time for transplantation purposes may be influenced by the specific culturing conditions for corneal transplants.

In European corneal banks, corneal tissue is stored for the purpose of transplantation mainly under organ culture conditions. The corneas are usually kept in culture at a temperature of 31–37 °C. Earle’s MEM (minimum essential medium) supplemented with fetal bovine serum and antibiotics or antimycotics is used as the medium. With the cold-store technique, which is primarily used in Anglo-American countries, the tissue is stored at 4 °C. The conditions of the cold storage technology do not correspond to the physiological conditions that the virus imposes on the host. Therefore, organ culture at 37°C corresponds to the physiological conditions required for successful cultivation of the virus. It should be noted that the storage conditions may affect the outcome of virus detection. We cannot conclude directly from organ culture, but it can be assumed that successful virus replication is even less likely with cold storage techniques. To further minimize or rule out the possible transmission of SARS-CoV-2 via corneal transplants, routine testing of corneal culture media or tissue samples could be considered in suspected cases prior to transplantation during the coronavirus disease 2019 (COVID-19) pandemic.

## Conclusions

In conclusion, this study adds further evidence supporting the very low probability of transmission of SARS-CoV-2 from an infected corneal graft donor to the recipient in corneal organ cultures under physiological conditions. Eye banking activities should take place in accordance with current eligibility criteria, ethical and legal implications for eye tissue donation and follow routine standard operating procedures, including iodine disinfection [2].

## Supplementary information

ESM 1 AE5. SI-1: Representative immunostaining analysis of AE5/K3 expression in cornea-derived epithelial cells. Magnification: x200, scale bar: 100μm. (PNG 1033 kb)

High resolution image (TIF 1402 kb)

ESM 2 CK12. SI-2: Representative immunostaining analysis of Cytokeratin 12 (CK12) expression in cornea-derived epithelial cells. Magnification: x100, scale bar: 100μm. (PNG 1092 kb)

High resolution image (TIF 1470 kb)

## Data Availability

The datasets used and/or analyzed during the current study are available from the corresponding authors on reasonable request.

## Abbreviations

SARS-CoV-2: Severe Acute Respiratory Syndrome-Coronavirus-2
COVID-19: Coronavirus Disease 2019
qRT-PCR: Quantitative Reverse Transcription-Polymerase Chain Reaction
HCMV: Human cytomegalovirus
ACE2: Angiotensin-converting enzyme 2
TMPRSS2: Transmembrane protease, serine 2
SARS-CoV-1: Severe Acute Respiratory Syndrome-Coronavirus-1
MERS-CoV: Middle East Respiratory Syndrome-Coronavirus
CDC: Center for Disease Control
FDA: Food and Drug Administration
BSL-3: Biosafety/protection Level 3
KM-1: Culture media 1
icSARS-CoV-2-mNG: Recombinant infectious SARS-CoV-2 clone expressing mNeonGreen
WT: Wild type
hpi: Hours post infection
GFP: Green fluorescent protein
Caco-2 cells: Colon-derived epithelial cells
MEM: Minimum essential medium
HCEC: Human corneal epithelial cells
MOI: Multiplicity of infection
M&M: Material and Methods Section
CK12: Cytokeratin 12
