# Supplementary figures and images for: Absence of detectable SARS-CoV-2 replication in ex vivo cultured cornea and cornea-derived epithelial cells
Source: Graefes Arch Clin Exp Ophthalmol. 2022 Aug 3;261(2):435–46. doi: 10.1007/s00417-022-05776-6 (PMC9362677; doi:10.1007/s00417-022-05776-6)

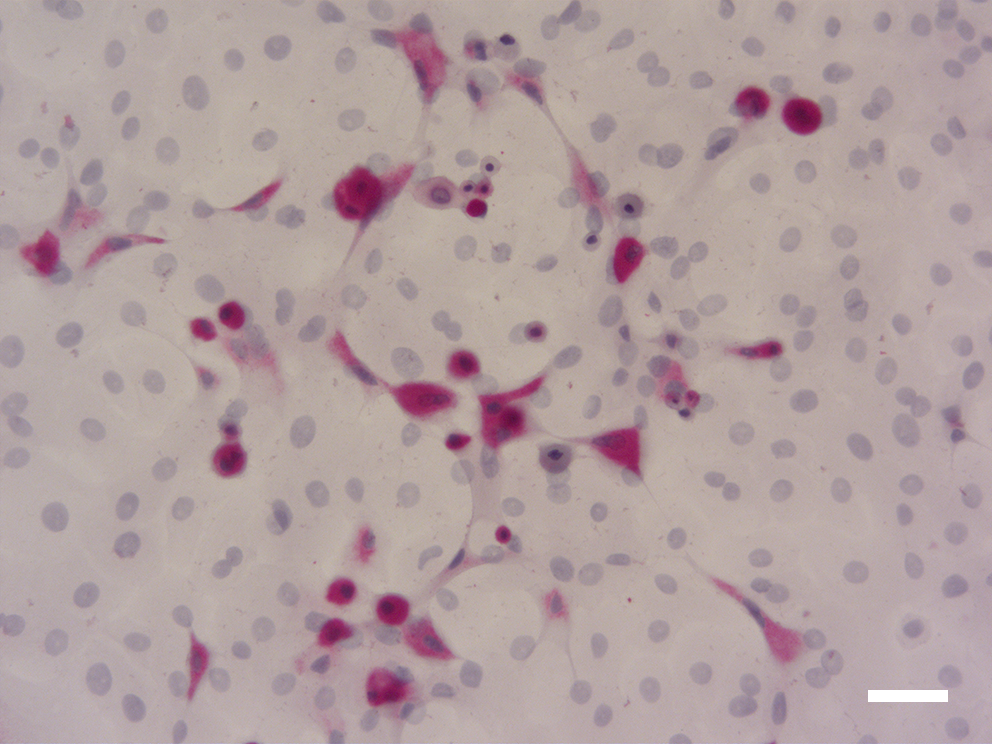

Supplement: Supplementary file 1 — AE5. SI-1: Representative immunostaining analysis of AE5/K3 expression in cornea-derived epithelial cells. Magnification: x200, scale bar: 100μm. (PNG 1033 kb) [file 417_2022_5776_Fig6_ESM.png]

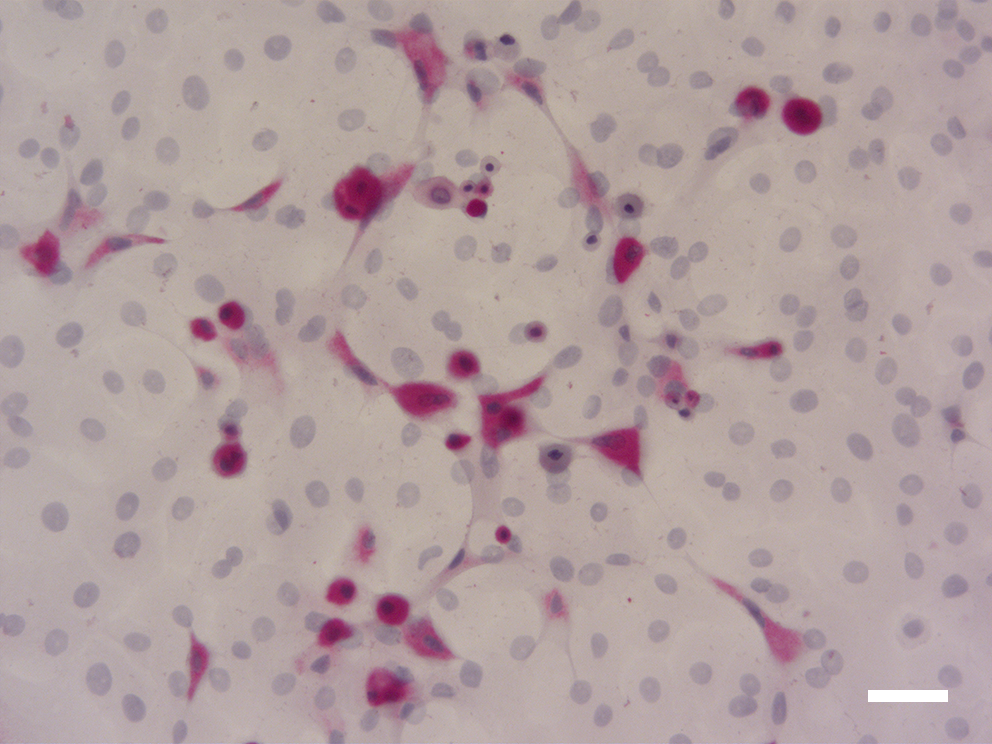

Supplement: Supplementary file 2 — High resolution image (TIF 1402 kb) [file 417_2022_5776_MOESM1_ESM.tif]

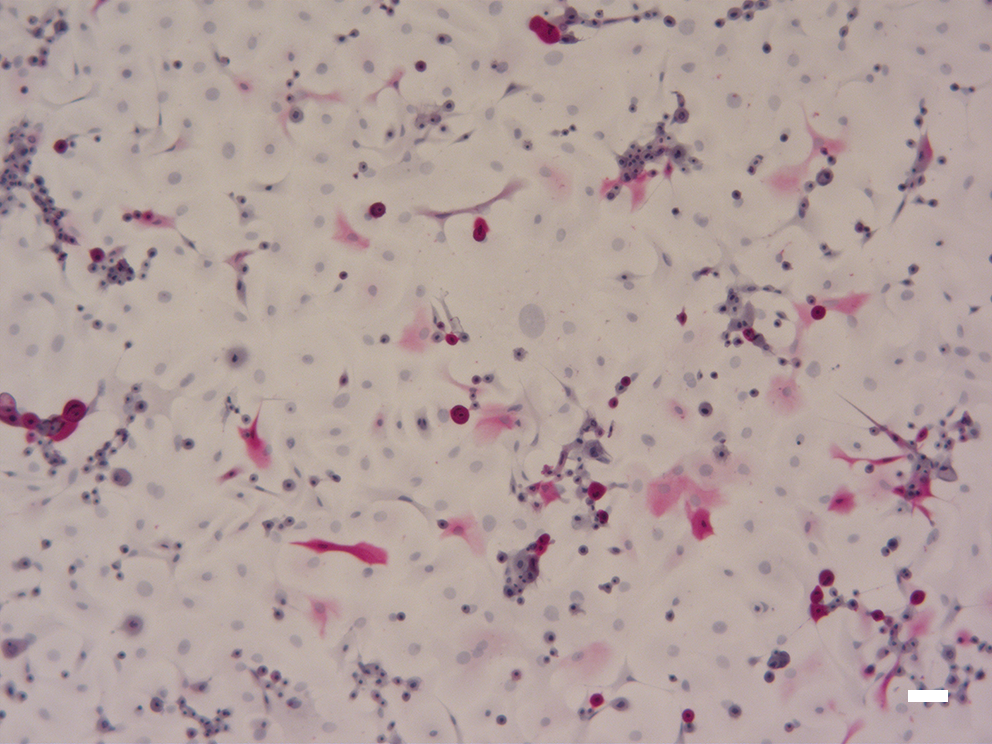

Supplement: Supplementary file 3 — CK12. SI-2: Representative immunostaining analysis of Cytokeratin 12 (CK12) expression in cornea-derived epithelial cells. Magnification: x100, scale bar: 100μm. (PNG 1092 kb) [file 417_2022_5776_Fig7_ESM.png]

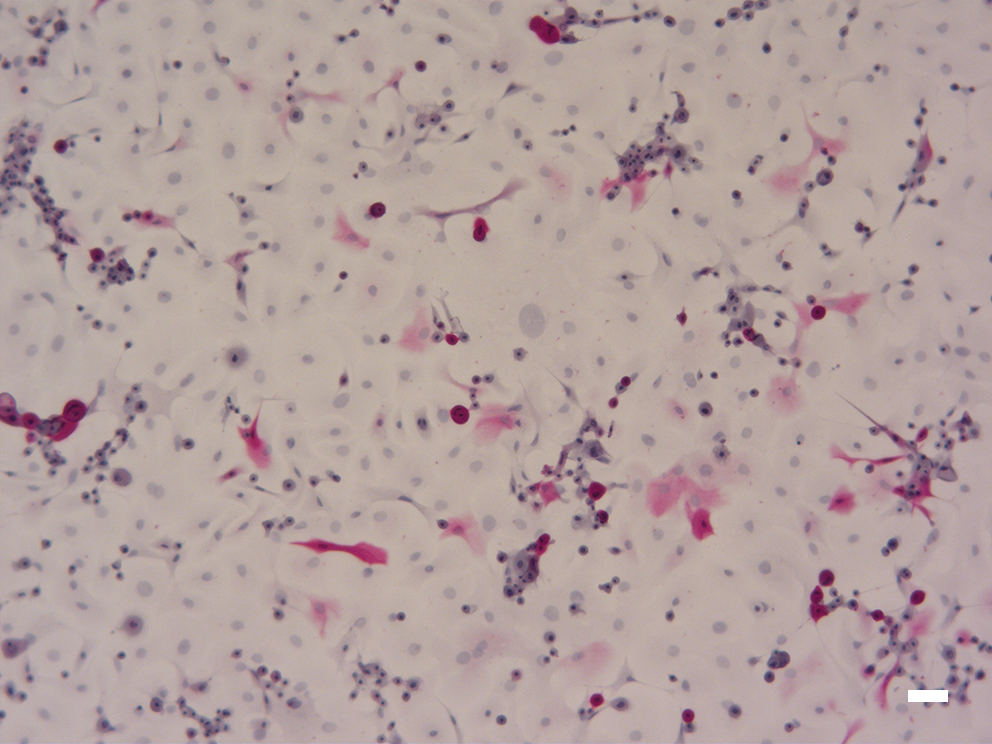

Supplement: Supplementary file 4 — High resolution image (TIF 1470 kb) [file 417_2022_5776_MOESM2_ESM.tif]
